# A nomogram for estimating intracranial pressure using acute subdural hematoma thickness and midline shift

**DOI:** 10.1038/s41598-020-77667-x

**Published:** 2020-12-11

**Authors:** Chun-Chih Liao, Heng-Chun Liao, Feipei Lai, Furen Xiao

**Affiliations:** 1 Department of Neurosurgery, Taipei Hospital, Ministry of Health and Welfare, Taipei, Taiwan; 2 School of Medicine, National Taiwan University, Taipei, Taiwan; 3 Department of Computer Science and Information Engineering, National Taiwan University, Taipei, Taiwan; 4 Department of Neurosurgery, National Taiwan University Hospital, Taipei, Taiwan

**Keywords:** Brain injuries, Computational models

## Abstract

Although criteria for surgical treatment of acute subdural hematoma (SDH) have been proposed, interaction exists between SDH, midline shift (MLS), and intracranial pressure (ICP). Based on our half sphere finite-element model (FEM) of the supratentorial brain parenchyma, tools for ICP estimation using SDH thickness (SDHx) and MLS were developed. We performed 60 single load step, structural static analyses, simulating a left-sided SDH compressing the cerebral hemispheres. The Young's modulus was taken as 10,000 Pa. The ICP loads ranged from 10 to 80 mmHg with Poisson's ratios between 0.25 and 0.49. The SDHx and the MLS results were stored in a lookup table. An ICP estimation equation was derived from these data and then was converted into a nomogram. Numerical convergence was achieved in 49 model analyses. Their SDHx ranged from 0.79 to 28.3 mm, and the MLS ranged from 1.5 to 16.9 mm. The estimation formula was log(ICP) = 0.614–0.520 log(SDHx) + 1.584 log(MLS). Good correlations were observed between invasive ICP measurements and those estimated from preoperative SDHx and MLS data on images using our model. These tools can be used to estimate ICP noninvasively, providing additional information for selecting the treatment strategy in patients with SDH.

## Introduction

Traumatic brain injury (TBI) is an important cause of mortality and severe disability. Subdural hematoma (SDH) represents a type of intracranial mass which is often diagnosed using a computed tomographic (CT) scan. “Acute SDH (aSDH)” is defined as an SDH diagnosed within days of TBI^1^. An SDH diagnosed later is termed as “subacute SDH” or “chronic SDH (cSDH)”. The “ellipsoid method” originally developed to calculate the volume of arteriovenous malformations^2^ has been widely applied in measuring the volume of post-traumatic masses, including SDHs^3^. Measurement of midline shift (MLS) and intracranial pressure (ICP) has also been advocated^1,4–6^. Details about MLS measurement and its automation were reviewed in our previous publication^5^.

Based on these quantitative evaluations, clear-cut SDH thickness (SDHx), MLS, and ICP thresholds for surgical treatment of aSDH have been proposed in existing clinical practice guidelines^1,6^. However, neurosurgeons have encountered numerous real-life counterexamples, such as patients who responded to non-surgical treatment despite large SDH thickness (SDHx) or those deteriorating rapidly despite relatively small SDHx. It has been shown that MLS in relation to acute SDHx predicts prognosis^7,8^. Disproportionately increased MLS compared to SDHx would raise concerns of brain tissue injury and edema, and hence, a higher ICP and a worse prognosis could be anticipated. Since interaction exists between SDHx, MLS, ICP, and other factors including the severity of brain atrophy^9^, a compound model including these variables is desirable. Using multiple regression, an ICP estimation equation derived from several CT features were proposed by Mizutani et al.^10^. It gives a reasonably accurate result but the measurement and calculation process is cumbersome and not possible at bedside or in low-resource settings.

We have constructed a half sphere model of the supratentorial brain using CT craniometric data from patients with brain concussion^11^. Using this geometric model and biomechanical parameters from the literature, we were able to simulate ICP changes associated with brain deformation caused by unilateral aSDH and cSDH using finite-element modeling (FEM)^12^. In this study, we carried out more simulations and then put these results into a lookup table to construct an estimation formula. Both the table and the formula can be used to estimate the ICP directly from SDHx and MLS on neuroimages such as CT or magnetic resonance imaging (MRI) of SDH patients. To simplify the estimation process, the complex formula was further converted into an easy-to-use nomogram^13^. With these tools, neurosurgeons can determine the treatment strategy without invasive and sometimes costly ICP measurement.

## Methods

Using our FEM of the supratentorial brain, we performed single load step, structural static analyses, simulating a left-sided SDH compressing the cerebral hemispheres from the outer surface of the left hemisphere under ANSYS ED 10.0 (ANSYS, Inc., Canonsburg, PA) software environment. The model assumes the shape of the upper half of a sphere with a diameter of 160 mm, with a volume of 1072 mL, which is very close to the average volume of our female subjects (1077 mL)^11^.

The length, width and height of this model are 160 mm, 160 mm and 80 mm, respectively. Each cerebral “hemisphere”, which assumes the shape of quarter sphere, has three surfaces: the convexity, the basal and the interhemispheric surfaces. The irregular basal surface is simplified and represented by a flat basal equatorial plane with a 40-mm circle removed from its center to simulate the midbrain occupying the tentorial incisura. To model the partially connected cerebral hemispheres, our model is divided into two compartments by the intact mid-sagittal plane (iMSP) representing the interhemispheric fissure. A semicircle 80 mm in diameter, located 20 mm anterior to the center of the basal plane, is removed from the iMSP to simulate the subfalcine space which allows brain herniation. The remaining part of the iMSP representing the falx is considered immobile. A detailed comparison between the geometry of our model and that of normal subjects has been reported previously^11^.

Using solid modeling, we generated the 3-D FEM of the supratentorial brain. The X, Y, and Z axes of the Cartesian coordinate system referred to the right-left, anterior–posterior, and superior-inferior anatomical axes, respectively. Because the model was only used to simulate deformation of the brain within the dura, it consists of only one type of linear elastic material: the brain parenchyma. The dura, the skull, and the scalp, necessary for modeling primary brain injuries, were omitted. The specific material properties of the brain are still controversial^14,15^. In our preliminary studies, the Young's modulus (YM) of the brain was taken as 10,000 Pa and the Poisson's ratio (PR) taken as 0.45^12^.

We chose the SOLID187 element to construct our model with 776 elements and 1391 nodes, which are points in the space whose position can be changed. This 3-D, 10-node tetrahedral element has a quadratic displacement behavior and is well suited to modeling irregular meshes^16^. Then, uniform compressive pressure loads were applied on the convexity surfaces of the left cerebral hemisphere, simulating increased ICP caused by the SDH. Displacements of the convexity surface of right cerebral hemisphere and the falx were constrained to 0 in all directions, simulating the rigid skull covering the brain and the tough dural infolding. Movement of the subfalcine nodes was unconstrained to simulate subfalcine herniation. On the basal surface, only nodes of the tentorial incisura were allowed to move in all directions, simulating transtentorial herniation. Other basal nodes of the brain were only allowed to move in X- and Y-directions over the skull base and displacements in Z-direction were constrained to 0.

Results from our preliminary studies showed a linear relationship between the ICP and the maximal brain deformation in all directions^12^. By altering the PR, we were able to reproduce realistic brain deformation patterns caused by aSDH and cSDH. Although we did not implement any external and internal structural details of the cerebral hemispheres including the sulci, the ventricles and the cisterns, the value of PR in our simplified FEM enables us to model the “tightness” of the whole supratentorial brain without them. Displacement of the model at the basal circumference was used to simulate SDHx and that at the basal midline simulating MLS. Values of SDHx and MLS were measured on the deformed model after applying different ICP loads to models with different PRs. Then, different ICP-PR combinations can be mapped into distinctive SDHx-MLS combinations.

Using factorial design, we performed 60 analyses consisting of 6 ICP conditions and 10 different PRs under ANSYS 10.0 ED environment. The tested ICP values were 10, 20, 30, 40, 60 and 80 mmHg, and the tested PR values were 0.25, 0.3, 0.35, 0.37, 0.39, 0.41, 0.43, 0.45, 0.47 and 0.49. A PR around 0.45 is considered as normal, values larger as “tight brain” and smaller as “atrophic brain”. The YM values in all experiments were kept as 10,000 Pa (about 75 mmHg). After collecting the SDHx and the MLS data from numerically stable results, we stored them in a lookup table. By finding the data point on the table with the smallest Euclidean distance to a given SDHx-MLS combination, the corresponding ICP-PR combination can be found. In this paper, we only collected ICP data in the lookup table and the PR data was omitted because it is not readily observable in clinical practice.

Applying multiple regression on original and on logarithmically transformed variables, we derived an ICP estimation equation from SDHx and MLS data. Then, the formula was transformed into a nomogram to allow easier ICP estimation without a computer or a calculator. Nomography is the graphical representation of mathematical relationships^13^. The simplest nomogram consists of three parallel straight axes, each representing a single variable appearing in an equation. A nomogram for determination of human body surface area from a subject’s height and weight has been used extensively^17^. To construct the ICP nomogram, the magnification factors on the axes as well as the distances between them were calculated from our estimation equation. After placing a straightedge on the completed nomogram across SDHx and MLS axes at known values measured on relevant images, the ICP is found as the value crossed on its axis.

To validate our simulation results, we retrospectively obtained the initial ICP readings from operation reports collected between Apr 2009 and Mar 2012 in our previous text mining study as the gold standard^18^. We routinely placed Codman MicroSensors (Johnson and Johnson Medical Ltd, Raynham, MA) to monitor the ICP in patients of severe TBI, including those with aSDH^19^. All documents containing both terms “subdural hematoma/subdural hemorrhage” and “intracranial pressure/ICP” were retrieved. These operation reports were then manually reviewed to retrieve SDHx, MLS data on preoperative CT images and ICP data recorded in each document. Those having no initial ICP measurements (i.e. only containing readings after hematoma removal or skin closure) were excluded. Patients with bilateral lesions, large parenchymal hematoma necessitating decompressive procedures were also excluded^20^. Manual ICP measurement data (ICPm) were then compared to ICP estimations derived from the lookup table (ICPt) and from the formula/nomogram (ICPf).

All methods were carried out in accordance with relevant guidelines and regulations. All experimental protocols and a waiver of consent were approved by National Taiwan University Hospital Research Ethics Committee (case number 201211002RIC).

## Results

### Simulation results

**Figure 1:**
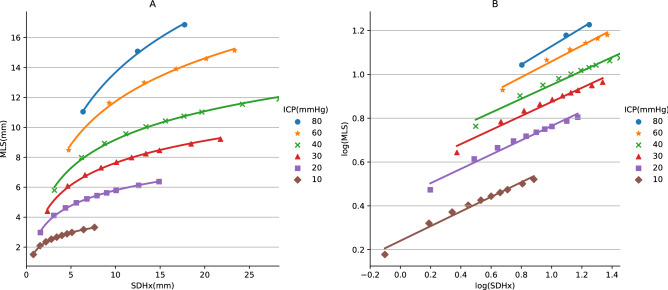
Scatter plots of simulated midline shift (MLS) against subdural hematoma thickness (SDHx) under different intracranial pressures (ICPs in mmHg) listed in legends, in linear (**A**), and in logarithmic scales (**B**). Units of SDHx and MLS in mm. Trend lines are generated from logarithmic (**A**) and linear (**B**) regressions using data points with the same ICP.

In the 60 analyses performed, 49 achieved numerical convergence. With higher ICP, convergence could not be achieved if the PR was too small, indicating excessive deformation of our FEM representing exceedingly soft or atrophic brain. With an ICP load of 60 mmHg, we could not obtain results with PRs of 0.25, 0.3, 0.35 and 0.37. With an 80-mmHg ICP, deformation results were only available with PRs of 0.45, 0.47 and 0.49. The values of SDHx and MLS from 49 successful simulation results were plotted on Fig. 1A; the values were also used to construct the lookup table.

The maximal SDHx derived from our model was 28.3 mm when the PR was 0.25 and the ICP was 40 mmHg. The maximal MLS was 16.9 mm when the PR was 0.45 and the ICP was 80 mmHg. By definition, the brain model does not deform without any pressure load, so both SDHx and MLS equal to 0 when ICP = 0. However, to create a lookup table and a formula, small values of SDHx and MLS were still required. Among our results, the minimal SDHx was 0.79 mm and minimal MLS 1.5 mm, both occurred when the PR was 0.49 and the ICP was 10 mmHg.

With the same PR, both SDHx and MLS assumed a linear relationship with ICP. Hence, the relationship between SDHx and MLS was also linear. Among our simulations, the ratio between MLS and SDHx was closest to 1 when the PR was 0.45. On the other hand, the relationship between SDHx and MLS was nonlinear with the same ICP when plotted in linear scale. It became linear after taking base-10 logarithmic transformation, as shown on Fig. 1B. The distances between these iso-ICP lines were not proportional to ICP, but also becomes so after taking logarithm. Based on these relationships, we derived the relationship between log(SDHx in mm), log(MLS in mm) and log(ICP in mmHg) using multiple linear regression:

$$log\left(ICP\right)= 0.614-0.520 log\left(SDHx\right)+ 1.584 log\left(MLS\right).$$

Using exponentiation notations, the formula can also be rewritten as

$$ICP= 4.111\left({MLS}^{1.584}/{SDHx}^{0.520}\right).$$

The formula fitted very well to our simulation results, with a R^2^ of 0.992. When the ICP was 10 or 20 mmHg, ICPf always had errors smaller than 2 mmHg. With an ICP of 40 mmHg, ICPf underestimate 3.6 and 3.4 mmHg when the PR was 0.25 and 0.49, respectively. With even higher ICP, ICPf still fits our simulation data very well except when the PR was 0.49, simulating a very tight brain with transtentorial herniation having a greater contribution to overall brain deformation compared to subfalcine herniation.

**Figure 2:**
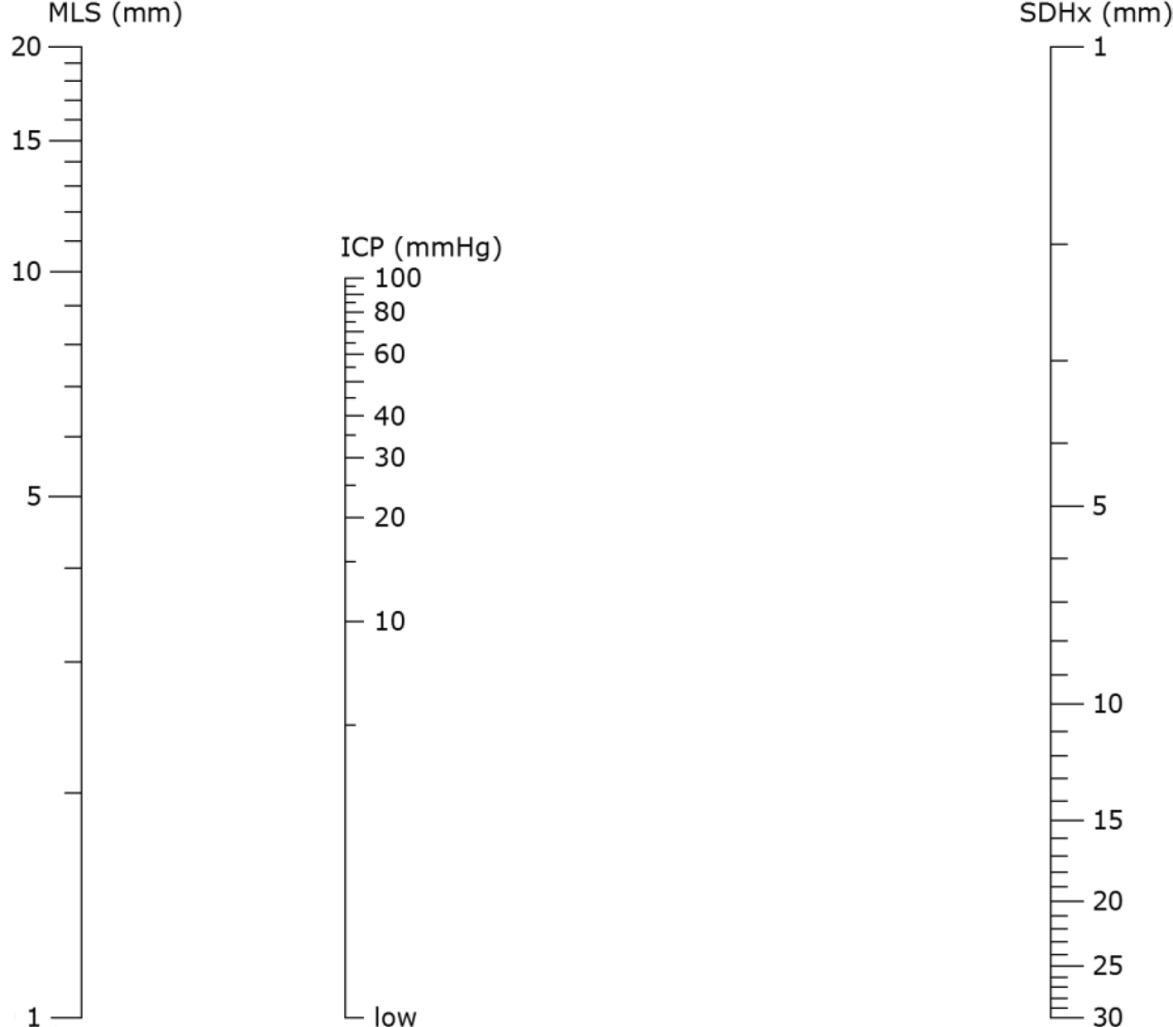
Nomogram for estimating the intracranial pressure (ICP) using subdural hematoma thickness (SDHx) and midline shift (MLS). The axes are shown in logarithmic scale.

To transform the formula into a nomogram shown in Fig. 2, we set the range of SDHx from 1 to 30 mm and that of MLS from 1 to 20 mm. These ranges are large enough to cover all data points measured in our previous studies^21^. Because the axes in the nomogram have logarithmic scales, the lowest values of both SDHx and MLS were set to 1 to avoid calculation errors. The full range of the ICP axis on the nomogram calculated from our exact formula is between 0.70 to 473 mmHg, the latter being clearly beyond pathological levels. Therefore, we limited the range of the ICP axis from 1 (denoted as “low” on Fig. 2) to 100 mmHg to cover our simulation results. The magnification factors for the SDHx, ICP, and MLS axes, all in logarithmic scale, are calculated as 291, 212 and 781, respectively. The relative distance between the ICP axis and the MLS axis was 291, and that between the ICP axis and the SDHx axis 781, indicating a larger influence of MLS on ICP. In addition, the origin of the SDHx axis is opposite from those of the MLS and the ICP axes, reflecting lower ICPs and larger hematoma volumes in atrophic brains when the MLS is the same.

### Validation of the lookup table and the nomogram

We retrieved 94 operation reports of 91 patients containing the desired terms from our database. Among them, 70 did not have the initial ICP reading and could not be used to verify our formula, including all 3 reoperations. In the remaining 24 patients, 6 were excluded because of the existence of large traumatic parenchymal hematoma and 2 were excluded for bilateral lesions. The SDHx, MLS, and ICPm recorded in the remaining 16 operation reports were used to validate our simulation results. All patients were adults having aSDH. Their SDHx ranged from 2 to 26 mm with an average of 10.6 ± 5.5 mm, the MLS ranged from 0.5 to 21 mm with an average of 8.0 ± 5.4 mm, and the ICPm ranged from 3 to 54 mmHg with an average of 28 ± 14 mmHg.

**Figure 3:**
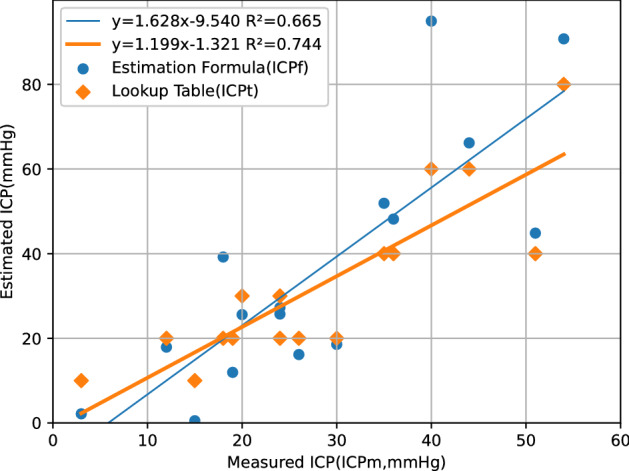
Comparison between intracranial pressures measured intraoperatively (ICPm) and those estimated from preoperative images using the lookup table (ICPt), and the formula (ICPf). Units in mmHg. The correlation between the ICPm and the ICPt (thick trend line) was better than that between the ICPm and the ICPf (thin line).

Using the SDHx and MLS data from each patient, we found the corresponding ICPt on the lookup table and calculated the ICPf using the formula. The ICPt ranged from 10 to 80 mmHg with an average of 33 ± 20. The errors ranged from −11 to 26 mmHg with absolute errors ranging from 1 to 26 (average: 8.8 ± 6.7) mmHg. The ICPf ranged from 0.5 to 95 mmHg with an average of 36 ± 28. The errors ranged from −14 to 55 mmHg. Compared to ICPf, ICPt correlated better with ICPm, as shown on Fig. 3. A total of 14 patients underwent decompressive craniectomies (DCs) after ICP monitor insertion. The ICPm, ICPt, and ICPf in the remaining 2 patients who did not proceed to hematoma evacuation or DC were both lower than 20 mmHg. Our formula performed better with lower ICPm, but tended to overestimate large ICP values. The two largest ICPf values, both larger than 90 mmHg, were 55 and 37 mmHg higher than the corresponding ICPm. Similarly, the largest error occurs with the only ICPt value of 80 mmHg (around upper right corner of Fig. 3).

## Discussion

We have proposed an FEM that can simulate brain compression by unilateral SDH. Based on this model, a formula for ICP estimation using SDHx and MLS was developed. Compared to previous regression formulas which were completely derived from measurements on real patient images^10^, ours was completely based on the FEM. The SDHx, MLS, and ICPm from our patients were used for validation instead. There are only two measurement variables in our formula, rather than five variables derived from more than a dozen measurements in previous ones. In addition to the geometry derived from CT craniometry, the only parameter that was kept constant was the YM. As shown in Fig. 3, setting the YM at 10,000 Pa seems to be a reasonable choice, as the slope of the ICPt-ICPm regression line was 1.12, close to being identical (Fig. 3). More intraoperative measurements are required to see whether this value is accurate.

In this study, the largest value of ICPt was set at 80 mmHg and that of ICPf set at 100. Both values are large enough for clinical applications. Although lower values of ICPm were recorded when the estimated ICPf was very high, this does not necessarily mean that our formula is inaccurate. When the ICP is extremely high, releasing some SDH from the small dural opening used for ICP monitor catheter insertion can cause rapid decompression with significant pressure reduction, resulting in a much lower ICPm than the real pressure. On the other hand, although our FEM can model cSDH and our tools do cover low-ICP ranges, they are less useful in guiding the treatment strategy of cSDH. The average pressure in cSDH is not high, and intracranial hypertension may not be the predominant mechanism underlying the neurological deficits observed in the cSDH patients^22^.

Our tools have several advantages. They are validated with real-life data of 16 patients and cost-free, allowing application in limited-resource situations and low-income countries. Ranges of SDHx and MLS are large enough to cover most clinical scenarios. Compared to the lookup table, the nomogram derived from the formula is even simpler and can be used anywhere without the need for any electronic devices. Anything resembling a straight line can be used as a straightedge on the nomogram printed on a piece of paper. By estimating the ICP non-invasively, neurosurgeons can do more with less. In TBI patients with multiple trauma, estimating the ICP also helps prioritizing treatment of different body regions. Although only validated using CT data, our results were based purely on anatomy and biomechanics and thus not imaging modality-specific. The lookup table and the formula can be used with SDHx and MLS measured on CT, MRI or even ultrasound studies^5^.

Our study has several limitations. Those related to geometric simplification has been described previously^12^. The FEM was based on the average head size of an adult and the resulting estimation is not applicable to pediatric patients. Our load condition simulated unilateral SDH. For bilateral SDH, the MLS is cancelled out, thus invalidating our formula. When using our lookup table or nomogram in SDH patients with coexisting parenchymal lesions, the laterality of these lesions can affect the accuracy of our estimations. SDH is frequently associated with parenchymal hematoma, and surgical strategy should consider both lesion types^1^. When the parenchymal hematoma and the SDH appear on the same side, the MLS is increased compared to that of “pure” SDH and the ICP also increases. Although only SDHx and MLS are used in our model, these additional ipsilateral parenchymal lesions may be considered as the compressed brain being tighter, and our tools may still be useful. Similar to bilateral SDH, our method becomes inaccurate when the parenchymal lesion is contralateral to the SDH.

Because our model estimates ICP from single time point CT, it does not account for the dynamic change of ICP. In acute settings, SDH, brain edema, MLS, and ICP can all progress fast over time. Repeated imaging or prompt surgical management should be considered on clinical deterioration.

## Conclusion

Based on our FEM, we have developed a lookup table, a formula, and a nomogram that can be used to estimate ICP in SDH patients with SDH using SDHx and MLS. These tools are cost-free and can be used anywhere to help refining the treatment strategy and they have been validated in aSDH patients. Further testing and calibration may further improve its usefulness in TBI patients.
